# Supplementary material for: Content-rich biological network constructed by mining PubMed abstracts
Source: BMC Bioinformatics. 2004 Oct 8;5:147. doi: 10.1186/1471-2105-5-147 (PMC528731; doi:10.1186/1471-2105-5-147)
Supplement: Additional File 2 — The original results of the above study (non-essential files are deleted to keep the file size under the limit set by BMC bioinformatics). [file 1471-2105-5-147-S2.bz2 › chilibotAdditionalFile2/dip20/258ID2569363E1022/html/CDC13_CDC2.html]

 


 **CDC13** and **CDC2** 
  
Found 69 abstracts in PubMed, retrieved 20.  
 

 What does Google say? 
 PDF only 
| .edu only 

---

**Interactive relationship** (e.g. stimulation, inhibition, etc)

**Neutral relationship**- The G1 S transition is controlled by interactions of  **Cdc13**   **Cdc2**  and its stoichiometric inhibitor, Rum1.  Ref: 9652094 Biophys Chem, 1998
- Antibodies to B type cyclins  **Cdc13**  specifically immunoprecipitated  **Cdc2**   **Cdc13**  complexes with associated kinase activity from P. carinii extracts.  Ref: 10837370 Am J Respir Cell Mol Biol, 2000
- Nuclear  **cdc2**  YFP fluorescence oscillates with that of  **cdc13**  YFP cyclin.  Ref: 11683390 J Cell Sci, 2001
- A specific synthetic peptide antibody generated to P. carinii  **Cdc13**  further demonstrated differential  **Cdc2**   **Cdc13**  activity over the life cycle of P. carinii, with greater activity in cysts compared with trophic forms of the organism.  Ref: 10837370 Am J Respir Cell Mol Biol, 2000
- At the G2 M boundary, the Cdc25 protein phosphatase is activated to dephosphorylate the  **Cdc2**   **Cdc13**  complex, resulting in abrupt activation of  **Cdc2**  kinase activity and entry into mitosis.  Ref: 9552387 Prog Cell Cycle Res, 1996
- The meta anaphase transition is controlled by interactions between  **Cdc13**   **Cdc2**  and the anaphase promoting complex, which labels  **Cdc13**  subunits for proteolysis.  Ref: 9652094 Biophys Chem, 1998

**Non-interactive relationship** (e.g. studied together, co-existance, homology, etc.)

- In opposition to these cyclins the rum1 inhibitor, a protein present exclusively in G1, prevents premature activation of the  **cdc2**  cig2 and the  **cdc2**   **cdc13**  complexes until cells have reached the critical cell size required to pass Start and initiate a new cell cycle.  Ref: 9552380 Prog Cell Cycle Res, 1996
- Cdc18 overexpression induces DNA rereplication without mitosis, as does elimination of  **Cdc2**   **Cdc13**  kinase during G2 phase.  Ref: 9436991 Mol Biol Cell, 1998
- All events of the fission yeast cell cycle can be orchestrated by fluctuations of a single cyclin dependent protein kinase  [ **CDC2** ] , the  **Cdc13**   **Cdc2**  heterodimer.  Ref: 9652094 Biophys Chem, 1998
- This mitotic induction operates, at least in part, through activation of the normal G 2 M CDK  [ **CDC2** ]  complex  **Cdc13**   **Cdc2** .  Ref: 12093738 EMBO J, 2002
- To further study the regulation of the life cycle of P. carinii, we characterized the P. carinii B type cyclin termed  **Cdc13** , whose binding to  **Cdc2**  is necessary for kinase activity.  Ref: 10837370 Am J Respir Cell Mol Biol, 2000
- We show that in fission yeast the mitotic B type cyclin  **Cdc13**   **Cdc2**  kinase associates with replication origins in vivo.  Ref: 12419251 Cell, 2002
- A detailed mathematical model for the fission yeast mitotic cycle is developed based on positive and negative feedback loops by which  **Cdc13**   **Cdc2**  kinase activates and inactivates itself.  Ref: 10884416 Proc Natl Acad Sci U S A, 2000
- An mfr1 null mutant completes meiosis II but remains with high levels of  **cdc13**  and  **cdc2**  kinase activity and has considerably delayed spore formation.  Ref: 11493649 J Cell Sci, 2001
- Consistent with this hypothesis, we report that Cdc18 interacts with  **Cdc2**  in association with  **Cdc13**  and Cig2 B type cyclins in vivo.  Ref: 9436991 Mol Biol Cell, 1998
- To investigate the mechanism coupling growth protein synthesis with cell division, we examined the relationship between the tyrosine kinase Wee1 that inhibits  **Cdc2**   **Cdc13**  mitosis inducing kinase by phosphorylating it, and protein synthesis inhibition in fission yeast.  Ref: 11119724 FEBS Lett, 2000
- **Cdc2**  YFP and  **cdc13**  YFP are highly enriched on the spindle pole body of cells in late G2 or arrested at S phase.  Ref: 11683390 J Cell Sci, 2001
- Positive feedbacks are created by  **Cdc13**   **Cdc2**  dependent phosphorylation of specific substrates inactivating its negative regulators Rum1, Ste9 and Wee1 Mik1 and activating its positive regulator Cdc25.  Ref: 10884416 Proc Natl Acad Sci U S A, 2000
- These findings identify Cdc18 as a key target of  **Cdc2**   **Cdc13**  and  **Cdc2**  Cig2 kinases in the mechanism that limits chromosomal DNA replication to once per cell cycle.  Ref: 9436991 Mol Biol Cell, 1998
- Surprisingly, Csk1 also activated  **Cdc2**  in complexes with either  **Cdc13**  or Cig2 cyclins.  Ref: 10226032 Curr Biol, 1999
- The model explains how fission yeast cells can exit mitosis in the absence of Ste9  **Cdc13**  degradation and Rum1 an inhibitor of  **Cdc13**   **Cdc2** .  Ref: 10884416 Proc Natl Acad Sci U S A, 2000
- If cdc13p cannot be recognized by the anaphase promoting complex,  **cdc2**  YFP and  **cdc13**  YFP remain associated with the spindle.  Ref: 11683390 J Cell Sci, 2001
- In the model organism Schizosaccharomysces pombe, potentially active  **Cdc2**   **Cdc13**  kinase complex accumulates throughout the S and G2 phases of the cell cycle.  Ref: 9552387 Prog Cell Cycle Res, 1996
- In fission yeast, the length of the G1 phase depends on a precise balance between levels of positive cig1, cig2, puc1, and  **cdc13**  cyclins and negative rum1 and ste9 APC regulators of  **cdc2** .  Ref: 10679013 Mol Biol Cell, 2000
- A slow negative feedback loop is turned on during mitosis by activation of Slp1 anaphase promoting complex APC, which indirectly re activates the negative regulators, leading to a drop in  **Cdc13**   **Cdc2**  activity and exit from mitosis.  Ref: 10884416 Proc Natl Acad Sci U S A, 2000
- Interestingly, Cdc6 is an in vitro substrate for  **Cdc13**   **Cdc2**  and Cig1  **Cdc2** , but not for Cig2  **Cdc2**  associated kinases.  Ref: 10381393 J Cell Sci, 1999
